# Temporal trends of ischemic stroke attributable to high fasting plasma glucose in China from the global burden of disease study 2019

**DOI:** 10.3389/fendo.2024.1408691

**Published:** 2024-08-05

**Authors:** Liangchen Tang, Li Xie, Yixin Liu

**Affiliations:** The People’s Hospital of Danyang, Affiliated Danyang Hospital of Nantong University, Danyang, China

**Keywords:** ischemic stroke, high fasting plasma glucose, disease burden, age-period-cohort analyses, China

## Abstract

**Background:**

Currently ischemic stroke poses a serious disease burden globally, and high fasting plasma glucose is one of the important risk factors. The aim of this study was to investigate the disease burden of ischemic stroke due to fasting glucose during 1990-2019 in China, to estimate the effect of age, period, and cohort on the trend of ischemic stroke disease burden, and to predict the disease burden of ischemic stroke in 2020-2030.

**Methods:**

Ischemic stroke burden data were obtained by screening from the Global Burden of Disease Study 2019 (GBD 2019) database for high-risk populations in China. Annual average percentage change (AAPC) was calculated using the Joinpoint regression model to assess the trend of ischemic stroke burden between 1990 and 2019. Age-period-cohort models were introduced to estimate the independent effects of age, period, and cohort on ischemic stroke burden, and to predict the ischemic stroke burden in 2020-2030 based on Bayesian age-period-cohort models.

**Results:**

From 1990 to 2019, the number of ischemic stroke deaths due to high fasting plasma glucose in China continued to increase with an AAPC of 3.61. Trends in age-standardized incidence rates did not show statistical significance. In the age-period-cohort analysis, the age effect of ischemic stroke burden showed a continuously increasing trend over the study period. The period effect showed an overall favorable trend over the study period. The overall and cohort effects for males showed an overall increasing trend, whereas the cohort effect for females showed a decreasing trend after a decreasing trend for the 1945 birth cohort.

**Conclusions:**

This study found that ischemic stroke due to high fasting plasma glucose in China has generally fluctuated between 1990 and 2019, with a decreasing trend in recent years, and projections also suggest that it will continue to show a decreasing trend in the future. Age and period of birth were the main elements influencing the burden of disease, especially among the elderly and men. Policies should be used to promote the prevention of known risk factors and to strengthen health management for key populations.

## Introduction

Ischemic stroke is a disease caused by necrosis of brain tissue due to narrowing or occlusion of the arteries supplying blood to the brain and insufficient blood supply to the brain (1). Globally, China has the highest estimated risk of stroke and the risk continues to rise. In China, more than 70% of stroke cases are ischemic stroke (2). World Health Organization reports that ischemic stroke is the second leading cause of death in the world and the third leading cause of death in China (3). Diabetes and ischemic stroke are common conditions that often occur together. Additionally, diabetes is a serious public health problem in China. According to the International Diabetes Federation, there were about 140 million people with diabetes in China in 2021, and it is expected to reach 160 million by 2030 (4). Studies have shown that cardiovascular disease is the leading cause of death in diabetic patients. Ischemic stroke caused by hyperglycemia is a challenge for chronic disease management in China.

The Global Burden of Disease Study 2019 (GBD 2019) reported that stroke ranked third in both age-standardized mortality and disability‐adjusted life years (DALYs) rates for all 204 diseases attributable to high fasting plasma glucose (5). High fasting plasma glucose leads to approximately 1/5 deaths and DALYs of cardiovascular disease. High fasting plasma glucose, as a risk factor for several chronic non-communicable diseases, such as diabetes, cardiovascular diseases, and tumors, has been shown to affect the organism through multiple pathways (6). Studies have shown that elevated glycemic can cause endothelial damage, increased blood viscosity, development of atherosclerosis, and increased lipids in plaques (7, 8). However, there is still a gap in research on the prevalence of ischemic stroke due to hyperglycemia in the Chinese population. Therefore, we leveraged the data from the GBD 2019, an updated global descriptive epidemiologic assessment of the disease, to systematically estimate and predict the trend in the burden of ischemic stroke attributable to high fasting plasma glucose in China from 1990 to 2030.

## Methods

### Data resource

The data for this study were derived from the Global Burden of Disease Study 2019 (GBD 2019), a study initiated by the Institute for Health Metrics and Evaluation (IHME) to estimate the global burden of different diseases and injuries. The GBD 2019 database includes data on 369 diseases, injuries, and their 87 risk factors for 204 countries and territories in 7 super-regions and 21 regions, covering the period 1990-2019 (3). More detailed information can be found on the official website (https://ghdx.healthdata.org/). In this study, data on the disease burden of ischemic stroke attributable to high fasting plasma glucose in China were retrieved from the database as the study population. The GBD 2019 study defines ischemic stroke based on the International Classification of Diseases, version 10 (ICD10) codes, which are coded as I63.0-I63.9. The GBD 2019 study defines a serum fasting glucose measurement of 4.8-8.4 mmol/L as high fasting plasma glucose (9). All the data were classified into 13 age groups (25-29, 30-34, …, 80-84, 85+), and age-standardized rates were calculated based on the GBD 2019 standard population.

### Analysis of temporal trend

The analysis of the temporal trend of the ischemic stroke burden attributable to high fasting plasma glucose in China was based on a Joinpoint regression model (10). The Joinpoint regression model divides trends over a long time period into several sub-segments by identifying inflection points (Joinpoints) in the trend data. It then evaluates the overall trend after assessing the trend of each segment separately, using the annual percent change (APC) for each sub-segment trend and the average annual percent change (AAPC) for the overall trend. An APC and AAPC greater than 0 indicate an upward trend, while an APC and AAPC less than 0 indicate a downward trend. In this study, we used the Joinpoint software (version 4.9.1.0; National Cancer Institute, Rockville, MD, US) to calculate the temporal trend of the ischemic stroke burden attributable to high fasting plasma glucose in China from 1990 to 2019, with a P-value of less than 0.05 considered statistically significant.

### Age-period-cohort analysis and projection

The age-period cohort model (APC model) differs from traditional linear models in that it considers three dimensions simultaneously, estimating the effects of age, period, and birth cohort on the burden of ischemic stroke attributable to high fasting plasma glucose (11). The age effect describes the changes in an individual’s life cycle as they age; the period effect reflects the trend of change in the society as a whole or the impact of events on the population; and the cohort effect emphasizes the role of the birth cohort to which an individual belongs on their disease burden (12). In this study, we conducted an APC analysis of ischemic stroke disease burden attributable to high fasting plasma glucose in China from 1990 to 2019 using a web tool (https://analysistools.cancer.gov/apc/) (13). In this study, the relative risks of age, period, and cohort effects were estimated for the 25-29 age group, the 2000-2004 burden of disease, and the 1945 birth cohort, respectively, as the reference group.

Next, we fitted the available data based on the Bayesian age-period-cohort model (BAPC model) to predict the burden of ischemic stroke attributable to high fasting plasma glucose in China in 2020-2030, and the BAPC model was able to better resolve the linear dependence among the three effects compared with the traditional APC model (14). The population data for 1990-2019 in this study were obtained from the GBD 2019 estimates, the population data for 2020-2030 were obtained from the projections for population data in the GBD study, and the calibration for the mortality and DALY rates were calculated based on the GBD 2019 standard population. Projections in this study were based on past trends and did not take into account changes in risk factors and interventions. BAPC modeling was performed using the BAPC package in the R 4.3.1.

## Result

### Temporal trend in the burden of ischemic stroke attributable to high fasting plasma glucose by sex in China

**Table 1** shows the disease burden of ischemic stroke attributable to high fasting plasma glucose in China in 1990 and 2019. Overall, the cases of deaths increased from 60,400 in 1990 to 174,200 in 2019 (AAPC=3.61, *P*<0.001). In addition, ASMR increased from 9.94/100,000 in 1990 to 10.18/100,000 and ASDR increased from 182.62/100,000 to 192.34/100,000, but neither ASMR nor ASDR showed statistically significant changes, fluctuating upward and then downward fluctuations between 1990 and 2019 trend (**Supplementary Table 1**).

**Table 1:** Burden of ischemic stroke attributable to high fasting plasma glucose in China, 1990 and 2019.

	1990	2019	AAPC	*t*	*P*
Male					
Deaths (ten thousand)	3.01	10.28	4.19	11.01	<0.001
ASMR (per 100,000)	11.63	14.08	0.43	1.04	0.299
ASDR (per 100,000)	198.01	239.17	0.54	1.45	0.148
Female					
Deaths (ten thousand)	3.03	7.15	2.93	12.40	<0.001
ASMR (per 100,000)	8.91	7.51	-0.71	-3.22	0.001
ASDR (per 100,000)	172.08	155.46	-0.38	-1.43	0.152
Both					
Deaths (ten thousand)	6.04	17.42	3.61	11.43	<0.001
ASMR (per 100,000)	9.94	10.18	-0.02	-0.06	0.949
ASDR (per 100,000)	182.62	192.34	0.08	0.28	0.782

### The burden of ischemic stroke attributable to high fasting plasma glucose across different age groups in China

**Figure 1:**
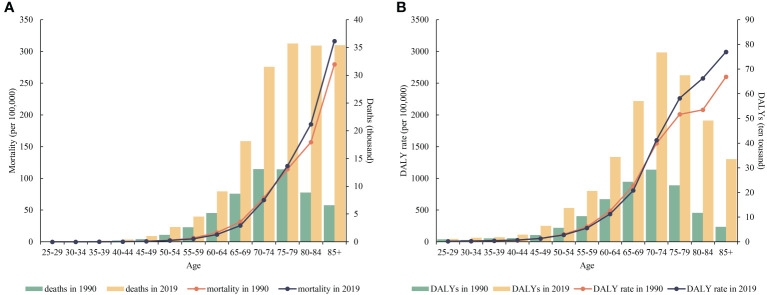
Burden of ischemic stroke attributable to high fasting plasma glucose in China, by age group, 1990 and 2019. **(A)** mortality (per 100,000) and deaths (thousand); **(B)**: DALYs rate (per 100,000) and DALYs (ten thousand).

**Figure 1** shows the burden of ischemic stroke attributable to high fasting plasma glucose among different age groups in China in 1990 and 2019. In general, the disease burden of ischemic stroke was concentrated in people aged 70 years or older. Compared with 1990, both the number of deaths and the number of DALY person-years increased substantially in 2019, especially in the older age groups. However, this trend did not appear in the mortality and DALY rates. In 2019, ischemic stroke mortality rates were lower than in 1990 for both the under-75 age group and higher than in 1990 for the over-75 age group, and similarly, DALY rates for ischemic stroke were lower than in 1990 for both the under-70 age group and higher than in 1990 for the over-70 age group in 2019.

### Age, period and cohort effects on the burden of ischemic stroke between 1990 to 2019

**Figure 2:**
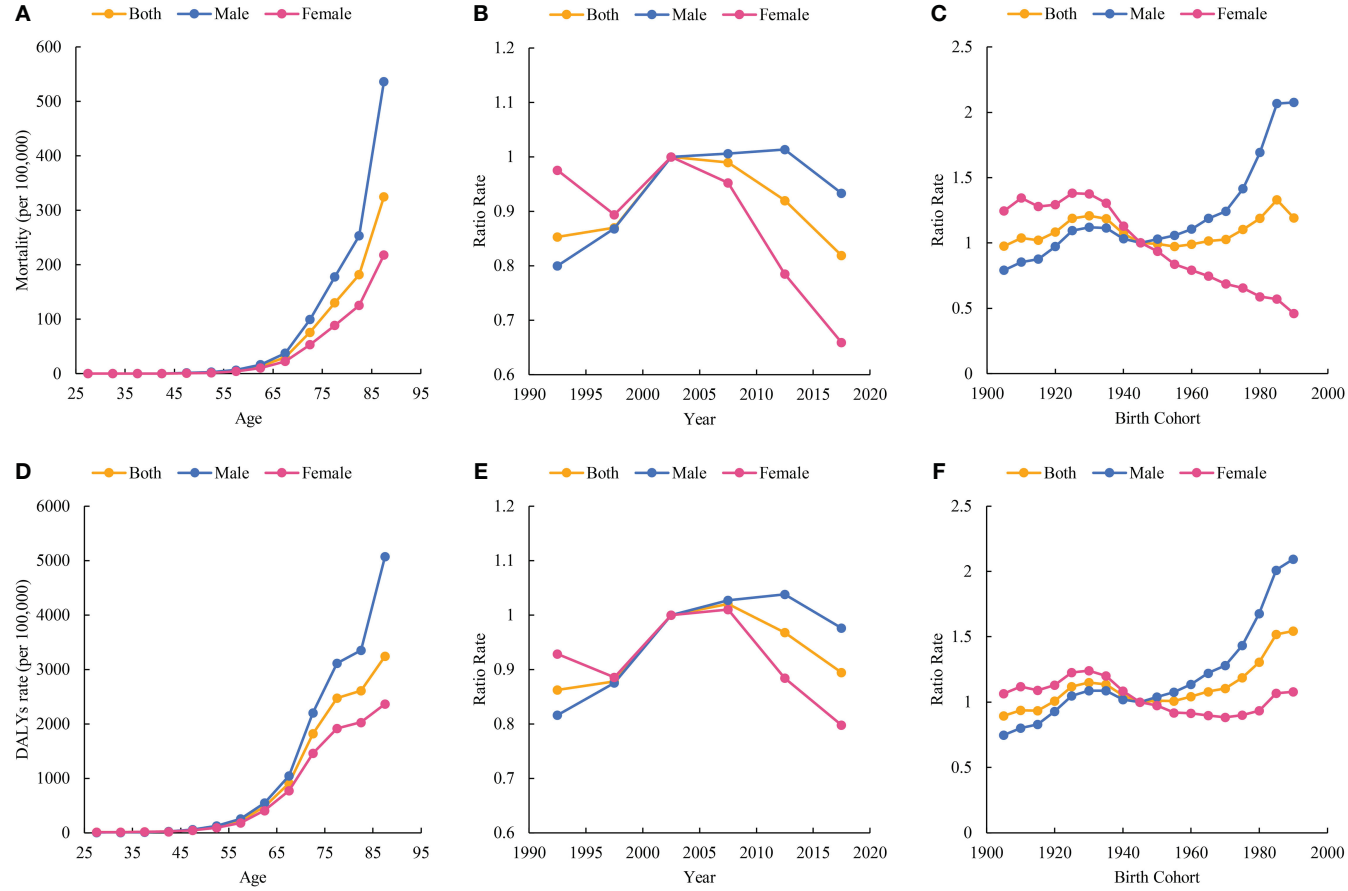
Parameter estimates of age, period, and cohort effects on burden of ischemic stroke attributable to high fasting plasma glucose and sex difference. **(A)** Age effect on mortality (per 100,000); **(B)** Period effects on mortality; **(C)** Cohort effects on mortality; **(D)** Age effect on DALYs rate (per 100,000); **(E)**: Period effects on DALYs rate; **(F)** Cohort effects on DALYs rate.

**Figure 2** shows the estimated age, period, and cohort effects for ischemic stroke mortality and DALY rates. The age effects of mortality and DALY rates increase linearly and peak in later years. The overall period effect for ischemic stroke mortality in 1990-2019 is favorable. However, the period effect for males shows an unfavorable trend over the period 2005-2014. The DALY rates for ischemic stroke show an overall favorable period effect from 1990-2019. However, the period effects both overall and for females showed unfavorable trends during 2005-2009, and for males during 2005-2014. The cohort effects for mortality and DALY rates showed fluctuating upward trends overall during 1990-2019. However, male and female burden cohort effects showed different trends. The disease burden for the male cohort effect showed a fluctuating increase and a surge among those born after 1945. For females, the disease burden cohort effect shows a fluctuating downward trend. Detailed information is shown in **Supplementary Tables 2**-**4**.

### The projected burden of ischemic stroke attributable to high fasting plasma glucose, 2020-2030

**Figure 3:**
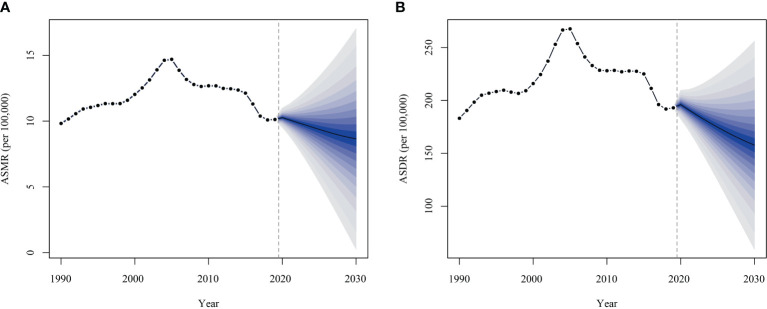
Projected burden of ischemic stroke attributable to high fasting plasma glucose in China, 2020-2030. **(A)** age-standardized mortality rate; **(B)** age-standardized DALYs rate.

According to the projections, the ASMR and ASDR for ischemic stroke attributable to high fasting plasma glucose in China are projected to show a similar and continuous decreasing trend from 2020 to 2030, as shown in **Figure 3**. Specifically, the ASMR is projected to decline from 10.26 per 100000 (with a 95% confidence interval of 9.52 to 11.00 per 100000) in 2020 to 8.66 per 100000 (with a 95% CI of 0.26 to 17.05 per 100000) and the ASDR from 196.07 per 100000 (with a 95% CI of 182.56 to 209.57 per 100000) to 157.68 per 100000 (with a 95% CI of 60.41 to 254.95 per 100000) in 2020, as detailed in **Supplementary Table 5**.

## Discussion

This study comprehensively analyzed the long-term disease burden of ischemic stroke attributable to high fasting plasma glucose in China from 1990 to 2019. We found that the number of deaths due to hyperglycemia-associated ischemic stroke decreased over 30 years, and age-standardized mortality and DALYs rates remained fluctuant. Notably, the burden rises sharply into old age, especially after age 70. The disease burden for females has shown the most significant decline in the last decade, while males show a more recent response. Predictions from the BAPC model suggested that the disease burden from hyperglycemia will continue to decline over the next 10 years. Against the background of an increasingly serious chronic disease management situation, stroke prevention as well as glycemic control are critical, particularly as the number of patients with diabetes continues to rise in China.

The AAPC analysis showed that age-standardized mortality and DALYs rate for ischemic stroke attributable to hyperglycemia remains stable over 30 years, which was in line with the temporal trend in the total burden of ischemic stroke (15). Compared with other major risk factors, high systolic blood pressure and ambient particulate matter pollution are the top 2 risk factors contributing to the burden and tend to be rising (15). This suggested that current diabetes management strategies in China were effective. A meta-analysis of 102 prospective studies showed that diabetes causes a twofold increase in the risk of multiple vascular diseases (16). Hyperglycemia induces an increase in inflammatory cytokines as well as monocytes and macrophages adhering to the endothelium, initiating oxidative stress and leading to endothelial dysfunction, sustained vascular injury, and ultimately atherosclerosis (17). High fasting glucose had also been demonstrated to cause pancreatic β-cell apoptosis, which reduced the effectiveness of glycemic control, leading to the development of diabetic complications (18). Given the huge burden of ischemic stroke in China, stroke care has become a national priority.

The China National Stroke Registry, initiated in 2007, completed a nationally representative stroke epidemiologic survey for 500,000 people in 2013. Additionally, the Phase 3 Stroke Survey Project, a multicenter, prospective, continuous, hospital-based registry study, began operations in 2015 to explore accurate early warning models for ischemic stroke and to assess healthcare delivery (19). The registry’s purpose is to develop strategies for continuous improvement of stroke care in China, using real-time data. The results indicate that China’s stroke incidence and mortality rates are among the highest in the world. Established in 2015, the National Stroke Center aims to have 3,000 hospitals join its network to promote stroke center construction and establish a regional emergency transport system for acute stroke (20). Studies have shown that the burden of stroke appears to grow more in rural areas (21), which may be related to differences in stroke awareness, quality of primary prevention, and socioeconomic status of the population. Improving stroke care and emergency transport capacity in rural hospitals is essential to reduce mortality from acute stroke attacks effectively. Considerable progress has been made in stroke care in China over the past decade, which corresponded to the declining trend in burden after 2010 in the period analysis. Prevention and control of ischemic stroke was a multifaceted and comprehensive collaborative process. China has emphasized the importance of glycemic control in both the investigation and treatment of stroke. However, significant gaps remain between guideline recommendations and clinical practice. Further emphasizing the role of glycemic control in stroke prevention could be beneficial.

Sex-based analysis showed a higher burden of ischemic stroke attributable to hyperglycemia in males than in females. Additionally, cohort effects analyses suggested an increasing trend of burden in males among individuals born after 1945, while the opposite trend was observed for burden in females. The burden attributed to hyperglycemia in males was often related to poor lifestyle habits, such as higher frequency of smoking and unhealthy diet. Some studies suggested that gender differences in ischemic stroke depend on the patient’s age, with the incidence of ischemic stroke being higher in males than in females during youth and middle age, and the incidence of ischemic stroke in females continued to increase after menopause (22). Although not identical to the findings of the present study, there is evidence that female patients have a worse functional prognosis for ischemic stroke and a reduced quality of life (23–25). National Health Interview Survey showed that the risk of diabetics suffering from cardiovascular disease was particularly acute in females (26). Another cohort study suggested that females with diabetes had a 27% higher risk of stroke than people with diabetes (27). Women tend to have a higher level of health consciousness (28), but diabetes seems to diminish or eliminate the female advantage and reveals that the burden on women should not be underestimated. Estrogen plays a protective role in many tissues, including the heart, brain, adipose tissue, and vascular system (29). With the onset of menopause and loss of estrogen, females should be aware of the risks for hyperglycemia-related ischemic stroke. Although the effects of diagnosis delay, inadequate treatment, and mechanisms of endothelial dysfunction linked to diabetes have been proposed (24), the drivers behind this gender difference remain largely unknown. Our study showed that males were still the main burden carriers, but the protection of females was also important. Recognition of the sex-specificity of stroke risk factors is an important way to move toward more effective and targeted stroke prevention strategies.

Age effects analysis indicated that the age-standardized mortality and DALYs rates increased rapidly with aging. As the aging population expands, the number of patients suffering from ischemic stroke will rise further (15). Age itself was an immutable and important risk factor for ischemic stroke, with differences in the effect of gender and diabetes mellitus (28). Diabetes serves as an independent risk factor for cardiovascular disease, and diabetes and ischemic stroke are two conditions that complement each other in terms of their cardiovascular risk implications. Both diseases cause inflammation, activate oxidative stress, induce endothelial damage, and promote cellular dysfunction and atherogenesis (17). Elderly stroke patients were long-term exposed to diabetes, which can cause chronic damage to the cerebral vasculature. Experimental stroke models also showed that chronic hyperglycemia led to cerebrovascular structural and functional defects (30). The elderly population was a heavily burden population, therefore early and ongoing screening for stroke and diabetes is essential.

Despite China’s tireless efforts in the care and prevention of ischemic stroke over the past decade, the epidemic has not been halted In contrast, the disease and economic burden of diabetes in China has risen rapidly over the same period (31, 32). However, the BAPC model showed burden of ischemic stroke attributable to high fasting plasma glucose declined consistently over the next 10 years. These phenomena suggested that current stroke and diabetes management strategies in China were working. Over the last 30 years, China has undergone significant changes in industrialization, demographics, and healthcare. There is already a general consensus among national health organizations and widespread public support for reducing the burden of stroke and diabetes. Early detection of the course of chronic disease, patient education, and regular checkups are effective measures for burden mitigation and inform public health practitioners and policymakers.

Although GBD 2019 has used rigorous algorithms for data estimation, this study still has some limitations. First, the data used in GBD 2019 were based on estimates and not on real observations. As a result, the estimates derived from these data modeling methods may be biased. Moreover, given the limited evidence available, there may still be potential risk factors that affect the available results. Second, the present study analyzed data at the national level and lacked data reflecting provincial and urban-rural differences. Using more disaggregated data would enable the identification of region-specific differences. Third, age-period-cohort analyses were conducted over multiple five-year periods, which may have obscured some of the subtle variations in age, period, and cohort effects. Finally, BAPC analyses do not account for possible variations in factors such as interventions and the environment, which can lead to some bias in the prediction results.

## Conclusion

In summary, we observed that ischemic stroke attributable to high fasting plasma glucose in China fluctuated overall from 1990-2019 and showed a decreasing trend in recent years and is projected to continue. Elderly people and men are the main groups affected by hyperglycemia. To reduce the burden of hyperglycemia-related ischemic stroke, the Chinese government should develop effective chronic disease management public health measures and policies to protect specific population groups.

## Data availability statement

The data that support the findings of this study are available from the Global Burden of Disease 2019 database, which is publicly available. Requests to access the database should be directed to https://ghdx.healthdata.org/gbd-2019.

## Ethics statement

Ethical approval was not required for the study involving humans in accordance with the local legislation and institutional requirements. Written informed consent to participate in this study was not required from the participants or the participants’ legal guardians/next of kin in accordance with the national legislation and the institutional requirements.

## Author contributions

LT: Conceptualization, Data curation, Methodology, Writing – original draft. LX: Data curation, Methodology, Writing – original draft. YL: Conceptualization, Writing – review & editing.
