# Supplementary material for: Temporal trends of ischemic stroke attributable to high fasting plasma glucose in China from the global burden of disease study 2019
Source: Front Endocrinol (Lausanne). 2024 Aug 5;15:1408691. doi: 10.3389/fendo.2024.1408691 (PMC11330829; doi:10.3389/fendo.2024.1408691)
Supplement: Supplementary file 1 [file DataSheet_1.pdf]

Supplementary Material

Supplementary Table 1 Burden of ischemic stroke attributable to high fasting plasma glucose in China from 1990 to 2019

Year	Deaths (ten thousand)	DALYs (ten thousand)	ASMR (per 100,000)	ASDR (per 100,000)
1990	6.04	135.26	9.94	182.62
1991	6.49	145.33	10.28	189.97
1992	6.98	156.04	10.68	197.81
1993	7.45	165.87	11.04	204.34
1994	7.75	172.09	11.16	206.24
1995	8.07	178.21	11.30	207.82
1996	8.41	184.24	11.45	209.06
1997	8.65	187.61	11.44	207.24
1998	8.94	191.95	11.46	206.00
1999	9.44	200.21	11.72	208.60
2000	10.18	213.27	12.18	215.39
2001	11.00	228.83	12.68	223.94
2002	12.04	250.18	13.29	236.63
2003	13.32	276.36	14.07	252.56
2004	14.54	300.52	14.80	266.13
2005	15.05	310.22	14.86	267.14
2006	14.63	303.34	14.02	253.20
2007	14.33	297.49	13.30	240.52
2008	14.34	296.32	12.90	232.33
2009	14.64	299.76	12.74	227.80
2010	15.17	308.27	12.78	227.31
2011	15.69	319.33	12.77	227.55
2012	16.04	329.28	12.56	226.16
2013	16.63	342.42	12.52	226.90
2014	17.20	355.15	12.42	226.58
2015	17.57	364.46	12.18	224.08
2016	17.07	356.14	11.35	210.46
2017	16.36	343.74	10.44	195.34
2018	16.60	349.77	10.15	191.20
2019	17.43	366.73	10.18	192.34

Supplementary Table 2 Age effects on burden of ischemic stroke attributable to high fasting plasma glucose between 1990 to 2019

	Mortality			DALYs Rate		
Age	Both	Male	Female	Both	Male	Female
25-29	0.05	0.04	0.09	7.70	5.20	11.97
30-34	0.08	0.08	0.09	8.59	7.31	10.78
35-39	0.17	0.17	0.18	14.57	13.14	17.07
40-44	0.35	0.42	0.29	22.91	24.75	20.96
45-49	0.98	1.16	0.79	53.22	58.84	47.26
50-54	2.34	2.87	1.78	109.51	125.99	91.56
55-59	5.26	6.56	3.88	220.29	254.79	183.47
60-64	13.42	16.54	10.13	479.20	546.31	408.96
65-69	30.19	37.82	22.37	909.57	1047.04	769.64
70-74	75.95	99.27	53.45	1822.91	2204.77	1457.49
75-79	130.21	177.62	88.30	2475.68	3114.30	1915.20
80-84	181.70	252.87	125.36	2607.01	3350.46	2027.79
85+	324.74	536.01	217.72	3239.79	5074.96	2364.17

Supplementary Table 3 Period effects on burden of ischemic stroke attributable to high fasting plasma glucose between 1990 to 2019

	Mortality			DALYs Rate		
Year	Both	Male	Female	Both	Male	Female
1990-1994	0.85	0.80	0.98	0.86	0.82	0.93
1995-1999	0.87	0.87	0.89	0.88	0.88	0.89
2000-2004	1.00	1.00	1.00	1.00	1.00	1.00
2005-2009	0.99	1.01	0.95	1.02	1.03	1.01
2010-2014	0.92	1.01	0.79	0.97	1.04	0.88
2015-2019	0.82	0.93	0.66	0.89	0.98	0.80

Supplementary Table 4 Cohort effects on burden of ischemic stroke attributable to high fasting plasma glucose between 1990 to 2019

	Mortality			DALYs Rate		
Median Year	Both	Male	Female	Both	Male	Female
1905	0.98	0.79	1.24	0.89	0.75	1.06
1910	1.04	0.85	1.34	0.94	0.80	1.12
1915	1.02	0.88	1.28	0.94	0.83	1.09
1920	1.08	0.97	1.29	1.01	0.93	1.13
1925	1.19	1.09	1.38	1.12	1.05	1.23
1930	1.21	1.12	1.37	1.15	1.09	1.24
1935	1.18	1.11	1.30	1.14	1.09	1.20
1940	1.07	1.03	1.13	1.05	1.02	1.08
1945	1.00	1.00	1.00	1.00	1.00	1.00
1950	0.99	1.03	0.94	1.01	1.04	0.97
1955	0.97	1.06	0.84	1.01	1.08	0.92
1960	0.99	1.11	0.79	1.04	1.14	0.92
1965	1.02	1.19	0.75	1.08	1.22	0.90
1970	1.03	1.24	0.69	1.10	1.28	0.88
1975	1.10	1.42	0.66	1.19	1.43	0.90
1980	1.19	1.69	0.59	1.30	1.68	0.93
1985	1.33	2.07	0.57	1.52	2.01	1.07
1990	1.19	2.08	0.46	1.54	2.09	1.08

Supplementary Table 5 The projected burden of ischemic stroke attributable to high fasting plasma glucose between 2020 to 2030

Year	ASMR (per 100,000)	ASDR (per 100,000)
2020	10.26 (9.52 to 11.00)	196.07 (182.56 to 209.57)
2021	10.09 (8.86 to 11.31)	191.47 (172.86 to 210.08)
2022	9.91 (8.08 to 11.73)	187.00 (161.80 to 212.20)
2023	9.73 (7.22 to 12.23)	182.72 (149.97 to 215.47)
2024	9.55 (6.31 to 12.79)	178.63 (137.67 to 219.58)
2025	9.37 (5.36 to 13.39)	174.69 (125.05 to 224.33)
2026	9.20 (4.37 to 14.03)	170.87 (112.20 to 229.53)
2027	9.04 (3.37 to 14.71)	167.22 (99.26 to 235.19)
2028	8.89 (2.35 to 15.44)	163.82 (86.30 to 241.33)
2029	8.77 (1.32 to 16.22)	160.65 (73.36 to 247.95)
2030	8.66 (0.26 to 17.05)	157.68 (60.41 to 254.95)
